# Partial *rpoB* Gene Sequencing Identification and Probiotic Potential of *Floricoccus penangensis* ML061-4 Isolated from Assam Tea (*Camellia sinensis* var. *assamica*)

**DOI:** 10.1038/s41598-019-52979-9

**Published:** 2019-11-12

**Authors:** Patthanasak Rungsirivanich, Angkhana Inta, Yingmanee Tragoolpua, Narumol Thongwai

**Affiliations:** 1 Department of Biology, Faculty of Science, Chiang Mai University, Chiang Mai, 50200 Thailand; 2 Graduate School, Chiang Mai University, Chiang Mai, 50200 Thailand; 3 Center of Excellence in Bioresources for Agriculture, Industry and Medicine, Chiang Mai University, Chiang Mai, 50200 Thailand

**Keywords:** Applied microbiology, Bacterial genetics, Food microbiology

## Abstract

Assam tea or Miang is a local name of *Camellia sinensis* var. *assamica* in northern Thailand. By the local wisdom, Assam tea leaves are used as the raw material in tea fermentation to produce “Fermented Miang” consumed by people in northern Thailand and the countries nearby. In this study, twenty-eight bacterial isolates were obtained from Assam tea leaf samples collected from Nan province, Thailand. Bacterial isolates were identified within 6 genera including *Bacillus*, *Floricoccus*, *Kocuria*, *Lysinibacillus*, *Micrococcus* and *Staphylococcus*. Among these, the strain ML061-4 shared 100.0 and 99.4% similarity of 16S rRNA and *rpoB* gene sequence with *F*. *penangensis* JCM 31735^T^, respectively. This is the first discovery of *F*. *penangensis* in Thailand. *F*. *penangensis* ML061-4 exhibited probiotic characteristics including lactic acid production (9.19 ± 0.10 mg/ml), antibacterial activities (*Escherichia coli* ATCC 25922 and *E. coli* O157:H7 DMST 12743), acid and bile salt tolerance (71.1 and 54.9%, respectively), autoaggregation (97.0%), coaggregation (66.0% with *E. coli* O157:H7), cell surface hydrophobicity (90.0%), bacterial adhesion (82.9% with *Lactobacillus plantarum* FM03-1), competitive inhibition (17.8% with *E. coli* O157:H7) and competitive exclusion (34.9% with *E. coli* O157:H7). Overall, the data suggested that *F*. *penangensis* ML061-4 had a great potential to be a probiotic.

## Introduction

*Floricoccus penangensis* is a bacterium in the family Streptococcaceae and has been classified as lactic acid bacteria (LAB). The genus was first described by Chuah *et al*.^1^ as it was clearly distinguished from the genera *Streptococcus* and *Lactococcus*. The sequence similarities of the 16S rRNA gene found closely related to lactococcal and streptococcal relatives, 92–94%. Currently, there is no report about discovery of *Floricoccus penangensis* in Thailand as well as their property reports.

More than two decades, the 16S rRNA gene sequence has been used to identify microorganisms as well as to decide a novel microbe (less than 97 and 95% sequence similarity for different species and genus, respectively)^2^. Nevertheless, many scientists have indicated that using of 16S rRNA gene are not applicable to multiple genera in taxonomic analysis. The 16S rRNA gene may not be effectively multiplied due to differential primer affinity and GC content^3^. Besides, it also demonstrates low resolution among closely related species^4^. The *rpoB* gene encodes the beta subunit of bacterial RNA polymerase. Its sequence is more discriminative than the 16S rRNA gene to distinguish various species of bacteria owing to the divergence levels of *rpoB* gene sequence that is explicitly higher than 16S rRNA gene^5^. Moreover, the partial *rpoB* gene sequence exhibits the precise reading frame leading to the easy verification of sequence accuracy. Therefore, the *rpoB* gene is a strong tool for bacterial identification^6^.

*Camellia sinensis* var. *assamica* or Assam tea or “Miang” is an indigenous perennial plant found in highlands of northern Thailand for a long time^7^. By local wisdom, fresh Assam tea leaves are fermented to produce a chewing refreshment product called “Fermented Miang”^8^. Assam tea can generally be found in upper northern Thailand including Chiang Mai, Lampang, Chiang Rai, Phayao, Nan and Phrae provinces^9^.

Probiotics are defined as live microorganisms that provide health benefits on the host when consumed in adequate amounts^10^. Probiotics can improve gut microbiota composition especially LAB, which have been suggested for health benefits including defense function as well as the nourishment of gastrointestinal tract in the body^11^. LAB are non-pathogenic bacteria found in fermentation for preservation, food and vitamins production^12^, help to improve microbial imbalance in the body caused by many factors including antibiotic treatment^13^ and are considered as generally recognized as safe (GRAS). Some LAB can produce bacteriocins, proteinaceous compounds with antibacterial activity against closely related strains^14^. LAB was classified within the phylum Firmicutes, which were divided into 14 genera: *Carnobacterium*, *Enterococcus*, *Floricoccus, Lactobacillus*, *Lactococcus*, *Lactosphaera*, *Leuconostoc*, *Melissococcus*, *Streptococcus*, *Oenococcus*, *Pediococcus*, *Tetragonococcus*, *Vagococcus* and *Weissella*^1,15^. This study aims to investigate the existence of microbes on Assam tea leaves, to demonstrate the use of partial *rpoB* gene sequence as a molecular tool for lactic acid bacteria identification and to evaluate probiotic property of the selected lactic acid bacteria found on Assam tea leaves. The results obtained will be useful for elucidating the involvement in tea fermentation process of microorganisms found on Assam tea leaves and will be further used in the food industry in many aspects such as functional ingredient and synbiotic production in the future.

## Results

### Sampling sites, Assam tea leaf collection and bacterial isolation

In this study, fresh Assam tea leaves were collected between 12 March 2016 and 24 March 2018 from three sampling sites in Pua (Code ML05 and ML06) and Phu Phiang (Code ML09) districts, Nan province, Thailand at an altitude ranging from 243 to 1,278 m above sea level. Meanwhile, fresh Assam tea leaves in Sakat sub district, Pua district (Code ML06) were collected twice on 12 March 2016 and 24 March 2018 (Table 1).

**Table 1:** Assam tea collecting site in Nan province.

Date obtained	No. of plant	Code	Location	Altitude (meter)	Province	No. of bacterial isolate per leaf	No. of species per leaf
12 Mar 16	1	ML051	19°12′0.76″N, 101°4′50.36″E	1,277	Pua sub district, Pua district, Nan	2	2
	2	ML052	19°12′0.96″N, 101°4′50.13″E	1,278	Pua sub district, Pua district, Nan	3	2
	3	ML061	19°15′53.62″N, 101°0′30.22″E	1,038	Sakat sub district, Pua district, Nan	4	4
	4	ML062	19°15′51.08″N, 101°0′31.03″E	1,030	Sakat sub district, Pua district, Nan	1	1
	5	ML063	19°15′49.09″N, 101°0′34.47″E	1,035	Sakat sub district, Pua district, Nan	2	2
24 Mar 18	6	ML064	19°16′3.63″N, 101°0′50.67″E	1,048	Sakat sub district, Pua district, Nan	2	2
	7	ML065	19°16′3.24″N, 101°0′51.85″E	1,055	Sakat sub district, Pua district, Nan	3	3
	8	ML066	19°16′7.22″N, 101°0′56.23″E	1,068	Sakat sub district, Pua district, Nan	3	3
	9	ML067	19°16′7.17″N, 101°0′58.05″E	1,085	Sakat sub district, Pua district, Nan	2	2
23 Mar 18	10	ML091	18°43′45.37″N, 100°49′57.38″E	243	Nam Kian sub district, Phu Phiang district, Nan	1	1
	11	ML093	18°43′45.25″N, 100°49′57.11″E	243	Nam Kian sub district, Phu Phiang district, Nan	1	1
	12	ML094	18°43′46.41″N, 100°49′55.32″E	243	Nam Kian sub district, Phu Phiang district, Nan	2	1
	13	ML095	18°43′46.24″N, 100°49′55.24″E	243	Nam Kian sub district, Phu Phiang district, Nan	1	1
	14	ML096	18°43′46.48″N, 100°49′54.21″E	243	Nam Kian sub district, Phu Phiang district, Nan	1	1

The data showed number of Assam tea plants, locations, altitude, number of isolate per sample, and number of species per sample.

Twenty-eight bacterial isolates with distinct colony morphology were isolated from fourteen fresh Assam tea leaves. The number of bacterial isolates collected from Pua, Sakat and Nam Kian sub districts were 5, 17 and 6 isolates, respectively. The Assam tea leaves collected from Sakat sub-district with the GPS coordinates of 19°15′53.62″N, 101°0′30.22″E (Code ML061) showed a maximum of four morphologically different bacterial colonies and had the highest number of bacterial isolates (Table 1).

### Bacterial identification

A total of 28 bacterial isolates obtained from 14 Assam tea plants, 10 isolates were identified as *Bacillus siamensis* with the 16S rRNA gene sequence similarity related to the type strain *B. siamensis* KCTC 13613^T^ between 99.7 and 99.9%. Three isolates had the 16S rRNA gene sequence related to *Staphylococcus haemolyticus* ATCC 29970^T^, 99.8–100% similarity. The other 15 isolates were identified as *B. altitudinis*, *B. aryabhattai*, *B. clausii*, *B. megaterium*, *B. subtilis* subsp. *subtilis*, *F. penangensis*, *Micrococcus aloeverae*, *S. cohnii* subsp. *cohnii*, *S. hominis* subsp. *hominis*, *S. hominis* subsp. *novobiosepticus*, *Kocuria halotolerans* and *Lysinibacillus contaminans*, under their 16S rRNA gene sequence similarity between 97.9 and 100.0%. Among these, 2 and 26 isolates were identified within Actinobacteria and Firmicutes phyla, respectively. The largest number of different species was obtained from Sakat sub district (Code ML061), 4 species per leaf, including *B. altitudinis*, *F. penangensis*, *M. aloeverae* and *S. haemolyticus* (Table 2; Supplementary Fig. S2).

**Table 2:** Bacterial species obtained from fresh Assam tea leaves, each bacterial isolate was compared with the type strain, the similarity (%), differences of the 16S rRNA gene sequence, and GenBank accession number.

Closest species	Type strain	Code	Similarity (%)	Differences in 16S rRNA gene sequence		GenBank accession no.
				Base substitution	Frameshift mutation	
*Bacillus siamensis*	KCTC 13613	ML064-2	99.9	1	—	MH236602
		ML065-2	99.9	1	1	MH236604
		ML066-2	99.7	4	2	MH236607
		ML067-2	99.9	1	—	MH236610
		ML091-2	99.9	1	1	MH236611
		ML093-4	99.9	1	1	MH236612
		ML094-2	99.9	1	—	MH236613
		ML094-6	99.8	2	—	MH236617
		ML095-3	99.9	1	—	MH236614
		ML096-3	99.9	1	—	MH236615
*Staphylococcus haemolyticus*	ATCC 29970	ML061-1	100.0	—	—	MH236595
		ML064-1	99.8	3	—	MH236601
		ML067-1	100.0	—	—	MH236609
*Bacillus aryabhattai*	JCM 13839	ML065-3	100.0	—	—	MH236605
		ML066-1	100.0	—	—	MH236606
*Micrococcus aloeverae*	DSM 27472	ML051-2	99.6	6	—	MH236592
		ML061-2	99.8	3	—	MH236596
*Staphylococcus hominis* subsp. *novobiosepticus*	GTC 1228	ML052-2	99.9	2	—	MH236616
		ML052-3	99.9	2	—	MH236594
*Bacillus altitudinis*	MTCC 7306	ML061-3	99.9	1	—	MH236597
*Bacillus clausii*	DSM 8716	ML062-2	99.6	5	1	MH236598
*Bacillus megaterium*	ATCC 14581	ML065-1	99.9	1	3	MH236603
*Bacillus subtilis* subsp. *subtilis*	JCM 1465	ML066-3	99.9	1	—	MH236608
*Floricoccus penangensis*	JCM 31735	ML061-4	100.0	—	—	MH050697
*Kocuria halotolerans*	YIM 90716	ML052-1	99.7	4	3	MH236593
*Lysinibacillus contaminans*	DSM 25560	ML063-2	97.9	29	—	MH236600
*Staphylococcus cohnii* subsp. *cohnii*	ATCC 29974	ML063-1	99.9	1	—	MH236599
*Staphylococcus hominis* subsp. *hominis*	DSM 20328	ML051-1	99.7	4	—	MH236591

Twenty-seven bacterial isolates showed high similarity of the 16S rRNA gene sequence between 99.6 and 100.0% that demonstrated the nucleotide substitution and frameshift mutation within the 16S rRNA gene sequence less than 6 and 3 positions, respectively. On the other hand, one bacterial isolate (Code ML063-2) had 97.9% similarity of 16S rRNA gene sequence when compared with the type strain *Lysinibacillus contaminans* DSM 25560^T^ with nucleotide substitution of 29 positions (Table 2).

### Strain characterization of *Floricoccus penangensis* ML061-4

**Figure 1:**
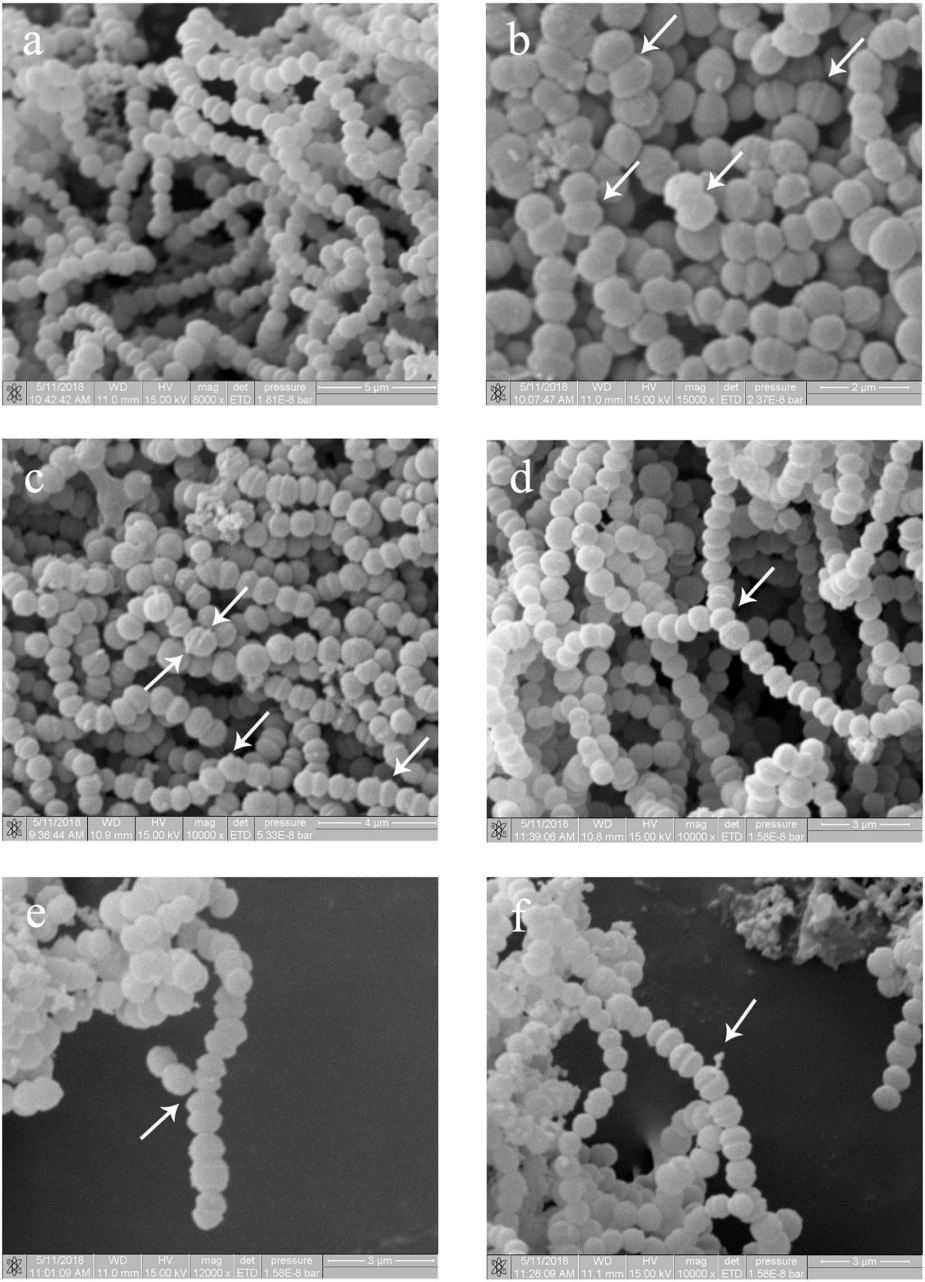
The SEM micrograph of *Floricoccus penangensis* ML061-4; (**a**) the morphological characteristic shows chain arrangement. (**b**) Arrows indicate cell division that occur diplococci. (**c**) Arrows indicate ridges on cells that might be due to recent cell division. (**d** and **e**) Arrows indicate branching filamentous, one of the two daughter cells undergo a second division that lead to a lateral branch filament formation. (**f**) Arrow indicates polysaccharide secretion.

*Floricoccus penangensis* ML061-4 was a Gram positive coccus, non-motile, non-spore forming, and negative for catalase, oxidase, indole and lysine decarboxylase tests. Their cell arrangement was found mostly in chains or occur as single cell and pairs. Some branching was occasionally observed. The SEM micrographs demonstrated that cell division occurred in one or two planes from a second division, producing diplococci and streptococci arrangement (Fig. 1). The strain ML061-4 produced acid from glucose (without gas), lactose, sucrose, mannitol, maltose and starch. It fermented raffinose to produce weak acid but could not ferment sorbitol. The starch was hydrolyzed but not for esculin. Positive results were obtained in Voges-Proskauer and methyl red tests. The growth temperatures were ranging between 10–40 °C. It could not grow in NaCl with concentration above 3% (w/v). The growth pH was between pH 5–8. Besides, the strain ML061-4 was able to grow in tryptic soy medium but not in MRS and BHI media.

**Figure 2:**
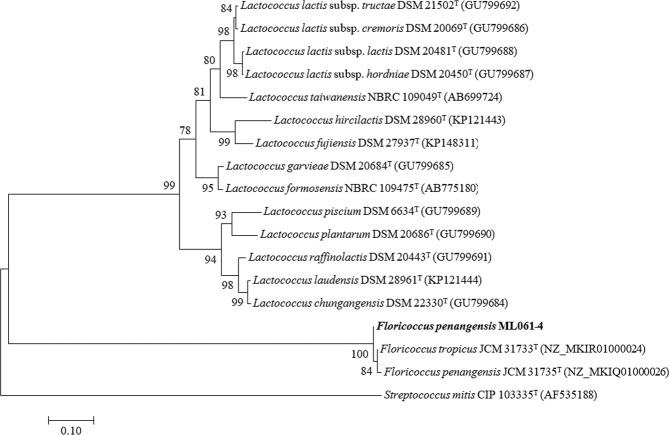
Phylogenetic relationships of the strain ML061-4 (bold), some species of the genus *Lactococcus* and *Floricoccus*, and related taxa based on *rpoB* gene sequence analysis. The branching pattern was generated by the neighbour-joining method. Bootstrap values (expressed as percentages of 1,000 replications). 50% are shown at the branch points. Bar, 0.10 substitutions per nucleotide position. *Streptococcus mitis* CIP 103335^T^ (GenBank accession no. AF535188) is presented as outgroup sequence.

The 16S rRNA (1,372 bp, GenBank accession no. MH050697) and *rpoB* (469 bp, GenBank accession no. MH183163) genes partial nucleotide sequences of strain ML061-4 were performed. Phylogenetic analysis based on the 16S rRNA indicated that the strain ML061-4 clustered with *F. penangensis*, supported by a bootstrap value of 93%. It had 100.0% sequence similarity (99% of query cover) with *F. penangensis* JCM 31735^T^ and 99.8% similarity (100% of query cover) with *F. tropicus* JCM 31733^T^. The *rpoB* gene sequence of strain ML061-4 had 99.6% sequence similarity with *F. tropicus* JCM 31733^T^, supported by a bootstrap value of 85%, and 99.4% similarity with *F. penangensis* JCM 31735^T^. The partial *rpoB* gene sequence of *F. penangensis* ML061-4 shared 49.5, 74.4 and 76.7% identity with *Streptococcus mitis* CIP 103335^T^, *Lactococcus plantarum* DSM 20686^T^ and *Lactococcus chungangensis* DSM 22330^T^ *rpoB* sequence, respectively. The phylogenetic tree of relationships based on the sequences of *rpoB* gene were analyzed using the 18 aligned sequences, the topology of the phylogenetic tree was supported by bootstrap values 1,000 replications which the main branch explicitly led to three branches of *Floricoccus* and *Lactococcus*, and *Streptococcus* genera (Fig. 2). The results indicated that *rpoB* gene could clearly distinguish between floricoccal, lactococcal and streptococcal groups. The finding showed that the *rpoB* gene fragment, 469 bp (positions 33 to 502 of *F. penangensis* JCM 31735^T^ *rpoB* gene sequence), contained hypervariable region and appropriated to use for group separation.

### Lactic acid determination

Lactic acid produced by *F. penangensis* ML061-4 was analyzed using HPLC. The total lactic acid was 9.19 ± 0.10 mg/ml consisting of 0.54 ± 0.07 mg/ml of D-lactic acid and 8.65 ± 0.03 mg/ml of L-lactic acid. The ratio of D:L was 1:16. The enzymes involved will be further elucidated.

### Evaluation of probiotic properties

#### Antibacterial activity and antibiotic susceptibility

The culture supernatant of *F. penangensis* ML061-4 could only inhibit growth of *E. coli* ATCC 25922 and *E. coli* O157:H7 DMST 12743 with the inhibitory clear zone of 7.0 ± 0.0 mm.

For the susceptibility evaluation, *F. penangensis* ML061-4 was susceptible to the testing antibiotics with the inhibitory clear zone ranging between 10.3 and 28.5 mm while *L. acidophilus* TISTR 2365 was susceptible to amoxyclav, ampicillin, ampicillin/sulbactum, cefixime, ceftriaxone, cefuroxime, cefuroxime axetil, cefotaxime, cefoxitin, gentamicin, meropenem, tetracycline and ticarcillin/clavulanic acid with the inhibitory clear zone of 9.9–49.0 mm but not for amikacin, co-trimoxazole and ofloxacin.

#### Acid-base tolerance

**Figure 3:**
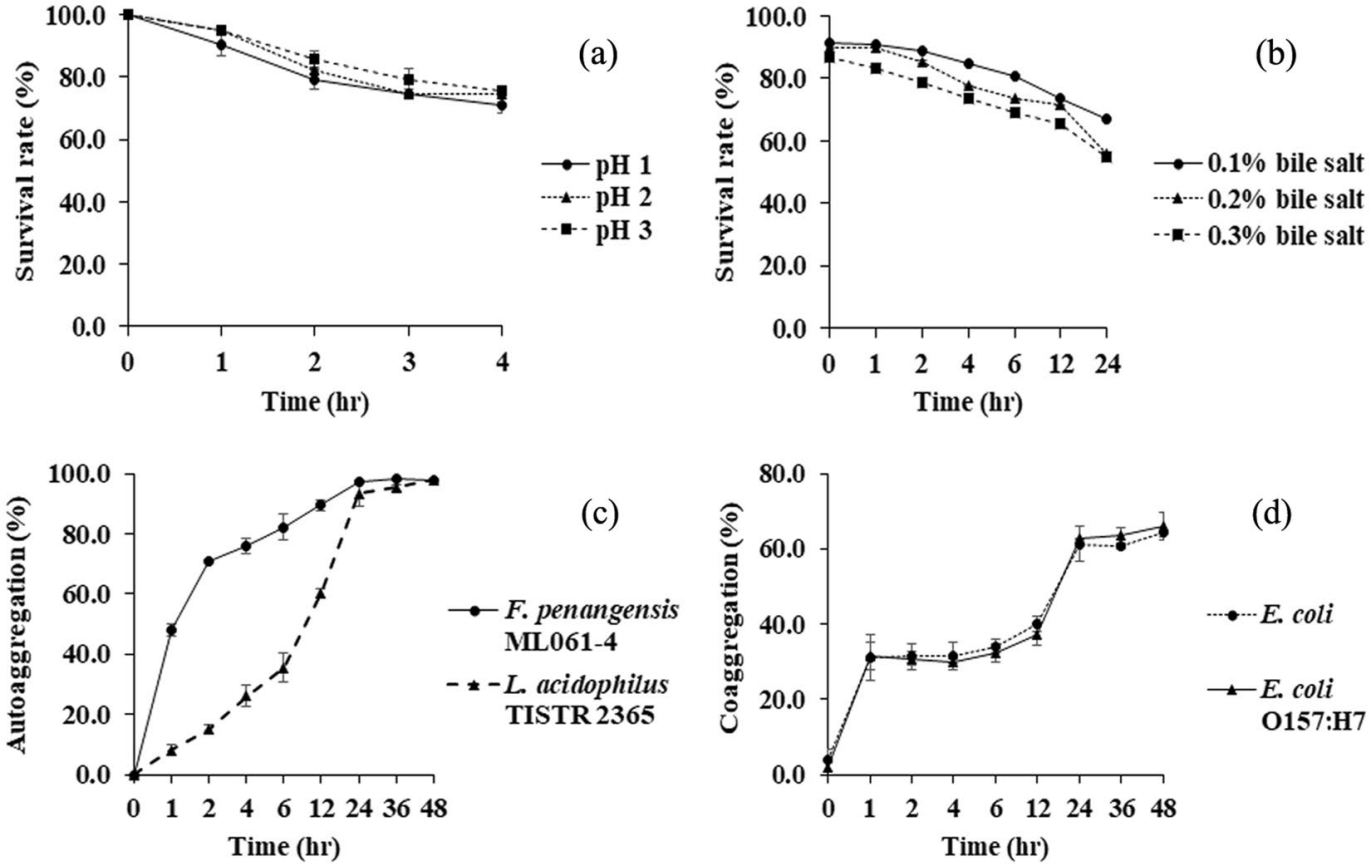
Survival rate of *F. penangensis* ML061-4 in acid (**a**) and bile salt (**b**). Ability of *F. penangensis* ML061-4 compared with *L. acidophilus* TISTR 2365 in autoaggregation (**c**) and coaggregation (**d**) with *E. coli* ATCC 25922 and *E. coli* O157:H7 DMST 12743. The experiments were triplicate conducted.

Survival rate of *F. penangensis* ML061-4 was higher than 70% after 4 hr of incubation in acidic pH, 1.0, 2.0 and 3.0. At pH 1.0, the survival rate was decreased gradually to 90.4, 79.4, 74.6 and 71.1% at 1, 2, 3 and 4 hr of incubation, respectively (Fig. 3a). Meanwhile, the survival rate of *F. penangensis* ML061-4 grown in TSB containing 0.1, 0.2 and 0.3% (w/v) of bile salt was higher than 50% subsequent to 24 hr of incubation. Once *F. penangensis* ML061-4 exposed to the mentioned media, the survival rate was decreased and maintained at between 54.9 and 66.9% after 24 hr (Fig. 3b).

#### Autoaggregation, coaggregation and cell surface hydrophobicity

*F. penangensis* ML061-4 and *L. acidophilus* TISTR 2365 had percentage of autoaggregation as 70.8 and 15.0% after 2 hr of incubation, respectively, significant (P < 0.05). Meanwhile, at 24 hr of incubation showed 97.0% and 93.2%, respectively, non-significant (p > 0.05) (Fig. 3c). Moreover, the ML061-4 demonstrated coaggregation ability with *E. coli* ATCC 25922 (64.6%) and *E. coli* O157:H7 DMST 12743 (66.0%) at 48 hr of incubation (P > 0.05) (Fig. 3d). Cell surface hydrophobicity of *L. acidophilus* TISTR 2365 was significantly (P < 0.05) lower than that of *F. penangensis* ML061-4, 27.5 and 90.0%, respectively.

#### Bacterial adhesion, competition and competitive exclusion

**Figure 4:**
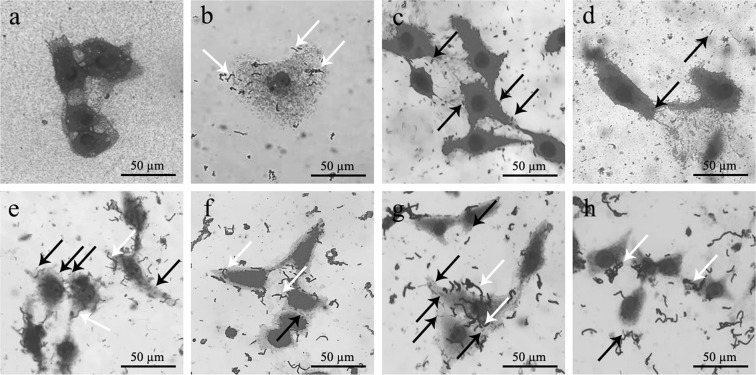
Bacterial adhesion of test bacteria on Vero cells. (**a**) Vero cells control; (**b**) *F. penangensis* ML061-4; (**c**) *E. coli* ATCC 25922; (**d**) *E. coli* O157:H7 DMST 12743. Bacterial competition on Vero cells (**e**) between *F. penangensis* ML061-4 and *E. coli* ATCC 25922; (**f**) between *F. penangensis* ML061-4 and *E. coli* O157:H7 DMST 12743. Competitive exclusion on Vero cells (**g**) between *F. penangensis* ML061-4 and *E. coli* ATCC 25922; (**h**) between *F. penangensis* ML061-4 and *E. coli* O157:H7 DMST 12743. White arrows indicate *F. penangensis* ML061-4 and black arrows indicate *E. coli* strains. Magnification: 400x.

Adhesion ability of *F. penangensis* ML061-4 was 21.4 and 82.9% when using *L. acidophilus* TISTR 2365 and *L. plantarum* FM03-1 as positive controls (100% adhesion), respectively, while *E. coli* O157:H7 DMST 12743 demonstrated as 5.4 and 20.9%, respectively (Table 3; Fig. 4a–d). In bacterial competition, *F. penangensis* ML064-1, *L. acidophilus* TISTR 2365 and *L. plantarum* FM03-1 inhibited the adhesion of *E. coli* O157:H7 DMST 12743 by 17.8, 15.1 and 14.4%, respectively, non-significant (p > 0.05) (Table 4; Fig. 4e,f). For competitive exclusion, all LAB strains showed displacement percentage to *E. coli* O157:H7 DMST 12743 between 34.9 and 38.4% (p > 0.05). Nevertheless, none of LAB strains tested was able to displace adhered *E. coli* ATCC 25922 (Table 4; Fig. 4g,h).

**Table 3:** Adherence of strain ML061-4, *E. coli* ATCC 25922 and *E. coli* O157:H7 DMST 12743 to epithelial cell as compared with *Lactobacillus* spp.

Bacterial strain	Adhesion (%)	
	*L. acidophilus* TISTR 2365	*L. plantarum* FM03-1
Control	100^b^	100^b^
*F*. *penangensis* ML061-4	21.4 ± 1.9^c^	82.9 ± 7.5^b^
*E. coli* ATCC 25922	127.2 ± 8.2^a^	493.6 ± 31.8^a^
*E. coli* O157:H7 DMST 12743	5.4 ± 0.5^d^	20.9 ± 2.0^c^

Data were expressed as mean ± standard deviation (n = 4). The difference alphabet was significantly different (p < 0.05) according to the Duncan’s multiple range tests.

**Table 4:** Competition between probiotic and pathogenic bacteria and competitive exclusion of pathogenic bacteria by probiotics to Vero cell.

Bacterial strain	Adhesion inhibition (%)		Pathogen displacement (%)	
	*E. coli*	*E. coli* O157:H7	*E. coli*	*E. coli* O157:H7
*F*. *penangensis* ML061-4	73.8 ± 3.3^a^	17.8 ± 6.8^a^	−24.6 ± 4^a^	34.9 ± 3.4^a^
*L. acidophilus* TISTR 2365	26.4 ± 2.9^c^	15.1 ± 2.7^a^	−74.5 ± 14.5^c^	36.3 ± 4.8^a^
*L. plantarum* FM03-1	56.8 ± 2.9^b^	14.4 ± 3.4^a^	−46.7 ± 9.3^ac^	38.4 ± 6.8^a^

Data were expressed as mean ± standard deviation (n = 4). The difference alphabet was significantly different (p < 0.05) according to the Duncan’s multiple range test.

## Discussion

Generally, Assam tea or Miang are found in highland forests, hillsides and high resourceful water areas^16^ at altitude of 450–1,500 m above sea level^8^. Interestingly, in this study Assam tea trees can grow at the elevation of 243 m above sea level (Phu Phiang district) which is lower than the previous report and also found cultivated within the community in the areas around villages indicating the close relationship between local people and Assam tea. Beyond the altitude above sea level that affects growth of tea, the climate, alkaline and acidic balance, soil nutrient as well as soil ecology also play a vital role in tea plant growth^17^. The sampling sites were both in the villages and on the mountains (over 1,000 m above sea level) where located far away from the community. Consequently, the existence of microorganisms on Assam tea leaves will bring about the role of microbes on Assam tea leaves in the tea fermentation process, first report in Thailand, especially lactic acid bacteria and their probiotic properties.

All bacterial isolates were member of Actinobacteria and Firmicutes phyla and were classified within 6 genera including *Bacillus*, *Floricoccus*, *Kocuria*, *Lysinibacillus*, *Micrococcus* and *Staphylococcus*. These bacteria have been widely found in nature which can be contaminated onto surface of leaves and stems^18–20^. *B. altitudinis*. *B. subtilis*, *K. halotolerans* and *M. aloeverae* were reported as endophytic bacteria involving in plant growth promotion and protection^21–24^. *B. clausii*, *B. megaterium*, *B. siamensis* and *B. subtilis* have been reported as probiotic. *Bacillus* spp. can form endospore and produce a bacteriocin-like substance, which help resisting stomach acidic condition and inhibiting pathogens^25–27^. *B. aryabhattai* produces vanillin from ferulic acid^28^, hence, it may involve in aroma and flavor occurred in fermented Miang. However, *S. haemolyticus*, *S. cohnii* and *S. hominis* are normal flora commonly live on human and animal skins^29–31^. Their existence on Assam tea leaves may be due to human activities as Assam tea growing area is located in the community.

LAB were known to involve in Assam tea or Miang fermentation^7^. Recently, Chaikaew *et al*.^32^ reported the diversity of LAB in fermented Miang. The prevalent species found were *L. plantarum*, *L. pentosus*, *L. panthesis* and *L. fermentum*. Furthermore, *Leuconostoc*, *Enterococcus*, *Pediococcus*, *Weisella* and *Lactococcus* were found as minor populations in fermented Miang. Interestingly, *Floricoccus* that has a close relationship with *Lactococcus* and *Streptococcus* was never reported. In this study, *Floricoccus* was found on fresh Assam tea leaves possibly due to some nutrients occurring on Assam tea leaves^33^ which enhances its survival. The existence of *Floricoccus* on an Assam tea leaf was not always as it was not found on all samples collected. The finding of LAB on Assam tea leaves could explain their key role in the tea fermentation process. Some reports proposed that the significant differences of bacterial communities in the environment may be related to soil compositions such as nutrients, acid and base conditions at the moment^34^. Hence, how *Floricoccus* exists on Assam tea leaves is to be further elucidated.

Reasonably, *Floricoccus* is not found in fermented Miang may be due to its lower ability to produce lactic acid than lactobacilli^35^ which is dominated in fermented Miang. Besides, this study also found that *Floricoccus penangensis* could not grow in MRS medium. These may be the reasons for the absence of *Floricoccus* species in fermented Miang. Some reports described that the non-growing LAB may associate with the high concentrations of polyunsaturated fatty acids in media which might inhibit growth^36^.

The *rpoB* gene has been usually used for identification when the 16S rRNA gene is unclear^5^. Identification of *Afipia, Bosea* and *Bradyrhizobium* spp. using *rpoB* gene showed the percentage cut-off between different genus ranging between 82 and 83%^37^. Thus far, there is no report about microorganisms on leaves of Assam tea. This is the first discovery report of *F. penangensis* in Thailand using the primer pair which is *rpoB* gene specific to identify bacteria in the genus *Floricoccus* that clearly separated from genera *Lactococcus* and *Streptococcus*.

The antibacterial activity of *F. penangensis* ML061-4 may lie within the amount of acid produced. Undissociated lactic acid could go through cell membrane and discharge hydrogen ion inside the cell leading to disturbance of important cell functions^38,39^. Furthermore, some species of LAB can produce bacteriocin or bacteriocin-like inhibitory substance (BLIS) that play a role in formation of bacterial cell membrane pore, inhibition of ATP synthesis and ultimately death of cells^40^. Notwithstanding, the inhibitory substance of *F. penangensis* ML061-4 will be further evaluated.

Antibiotic susceptibility is proposed as a vital criterion for potential probiotics to ensure that the antibiotic resistance gene will not be transferred to recipient bacteria in the gut which leads to the development of antibiotic-resistant bacteria^27^. Moreover, antibiotic resistance gene transfer also may be related to the change of microbial community in the environment including Assam tea growing area. In this study, *F. penangensis* ML061-4 was inhibited by all test antibiotics indicating its safety as a potential probiotics while *L. acidophilus* TISTR 2365 was sensitive to 13 of 16 antibiotics tested.

The acid tolerance of *F. penangensis* ML061-4 may be based on the histidine decarboxylation pathway, which generates proton motive force resulting in ATP synthesis^41,42^. Furthermore, the acid tolerance in LAB also related to glutamate decarboxylase system and arginine deiminase pathway^43^. Additionally, some reports suggested that bile resistance is associated with enzyme activity of bile salt hydrolase (BSH), which leads to the toxicity reduction^42^. However, the immediately reduction of *F. penangensis* ML061-4 in base condition at the beginning can be explained as a result of antibacterial activity of bile salt and its bacterial membrane ability^44^.

The autoaggregation and hydrophobicity assays are used for determination of adhesion ability of probiotics to intestinal mucosa, which is necessary for host gut bacterial colonization, and bacterial coaggregation can prevent pathogenic bacterial infection and colonization^45–47^. In this study, *F. penangensis* ML061-4 exhibited a higher autoaggregation and hydrophobicity than *L. acidophilus* TISTR 2365.

Bacterial adhesion onto epithelial cells is a considerable property of probiotic to reduce enteropathogenic adhesion and infection^48^. Bacterial cell wall components such as proteins, carbohydrates and fatty acids were responsible for the adherence to the intestinal mucosa^49^. High potential probiotics should demonstrate bacterial competition and competitive exclusion to disturb and interrupt pathogenic infection, colonization and biofilm formation as well as produce polymeric substances such as exopolysaccharides to help probiotic colonization within intestine^50,51^. In this study, *F. penangensis* ML061-4 represented the ability of adhesion, competition and displacement similar to *L. acidophilus* TISTR 2365 and *L. plantarum* FM03-1 that are considered the great probiotics. These characteristics are harbored by *F. penangensis* ML061-4 which has high potential to be a good probiotic.

## Methods

### Assam tea leaf collection

Fresh Assam tea leaves were obtained from Pua and Phu Phiang district, Nan province of Thailand. The source of Assam tea is presented in Table 1 and Supplementary Fig. S1. Each fresh Assam tea leaf surface was swabbed by a sterile cotton swab which was subsequently kept in 10 ml 0.85% (w/v) of NaCl prior to bacterial isolation within 24 hr.

### Bacterial isolation

Each sampling swab was serially diluted with 0.85% (w/v) of NaCl and spread on tryptic soy agar (TSA) (Merck™, Germany) prior to incubation at 37 °C for 24–48 hr. All pure bacterial isolates were stored at −20 °C in tryptic soy broth (TSB) (Merck™) containing 20% (v/v) of glycerol.

### Genomic DNA extraction and gene amplification

The chromosomal DNA of each strain was extracted by modification of the protocol described by Pitcher *et al*.^52^. The 16S rRNA gene was amplified using universal bacterial primers, 27F (5′-AGA GTT TGA TCM TGG CTC AG-3′) and 1492R (5′-TAC GGY TAC CTT GTT ACG ACT T-3′)^53^. The primers used for amplification of the housekeeping gene *rpoB* were RpoBLac1F (5′-TAC GGK AAA CAC CGT A-3′) and RpoBLac1R (5′-TCA ARC CAW GCT CCA CGG-3′), which were previously designed and reported by Meucci *et al*.^54^. The amplification cycles were initially denatured at 94 °C for 5 min, followed by 30 cycles of denaturation at 94 °C for 30 sec, annealing at 56 °C (16S rRNA) or 47.5 °C (*rpoB*) for 30 sec, extension at 72 °C for 1 min, final extension at 72 °C for 5 min, and kept at 4 °C. The PCR products were electrophoresed on 0.8% (w/v) agarose gel containing 1X nucleic acid staining solution (RedSafe^®^, iNtRON Biotechnology, Inc., Korea) for 50 min at 95 V 300 mA in 1X Tris-acetate-EDTA (TAE) buffer (40 mM Tris-acetate, 1 mM EDTA, pH 8.0) using electrophoretic gel system (EC320, Minicell Primo, USA) at room temperature. The 1 kb DNA Ladder (RBC Bioscience, Taiwan) was used as a marker. The gels were visualized under ultraviolet light by gel documentation system (SynGene, USA), and gel images were captured using monochrome CCD camera (TM-300, PULNiX, Japan). The amplicons were purified and sequenced by a DNA sequencing services (First BASE Laboratories Sdn Bhd., Malaysia). The 16S rRNA and *rpoB* gene sequence were performed by comparing with GenBank and EzBioCloud databases. The sequence data was aligned, and phylogenetic tree was analyzed by a neighbor-joining method^55^ with a MEGA 7 program^56^.

### Bacterial morphological, biochemical and physiological characterization

The strain ML061-4 was investigated its morphological characteristics. Scanning electron microscopy was performed as described by Arroyo *et al*.^57^ with slight modifications using the scanning electron microscope (SEM) (EFI Quanta 200 3D, USA). For biochemical characterization, various tests were conducted including carbohydrate fermentation (glucose, lactose, sucrose, mannitol, maltose, raffinose, sorbitol and starch), esculin and starch hydrolysis, indole, lysine decarboxylase, catalase and oxidase production, and Voges-Proskauer and methyl red tests. Various growth conditions were determined including growth in the presence of 3, 4 and 6% (w/v) NaCl, growth at various temperatures and pHs. The media used were de Man, Rogosa and Sharpe (MRS) and brain heart infusion (BHI) (Merck™).

### Lactic acid analysis

The *F. penangensis* ML061-4 was cultivated in TSB and incubated at 37 °C for 24 hr. The culture broth was centrifuged at 6,000 rpm 4 °C for 5 min and filtered by nylon membrane filter, 0.22 µm. The filtrate was kept at −20 °C before evaluation of lactic acid content. Lactic acid was determined using a high-performance liquid chromatography (HPLC) system (1200 series, Agilent Technologies, Inc., USA), equipped with a UV detector at 254 nm using an Astec CLC-L (150 × 4.60 mm) column (Sigma-Aldrich™, USA) with 0.005 M of CuSO_4_ as the mobile phase at a flow rate of 1.0 ml/min. The D- and L-lactic acids (Sigma-Aldrich™) were used as standards.

### Evaluation of probiotic properties of the strain ML061-4

#### Antibacterial activity

Agar well diffusion method was carried out according to the method of Bonev *et al*.^58^. The culture broth was tested against *Bacillus cereus* TISTR 687, *Escherichia coli* ATCC 25922, *E. coli* O157:H7 DMST 12743, methicillin resistant *Staphylococcus aureus* DMST 20625, *Pseudomonas aeruginosa* ATCC 27853, *Salmonella* Typhi DMST 22842, *Shigella dysenteriae* DMST 1511, *S. aureus* ATCC 25923 and *Vibrio cholerae* DMST 2873.

#### Antibiotic susceptibility test

The antibiotic susceptibility test of the strain ML061-4 was examined using antibiotic discs (Himedia^®^, India) including amikacin (30 µg), amoxyclav (30 µg), ampicillin (10 µg), ampicillin/sulbactum (10 µg/10 µg), cefixime (5 µg), ceftriaxone (30 µg), cefuroxime (30 µg), cefuroxime axetil (30 µg), cefotaxime (30 µg), cefoxitin (30 µg), co-trimoxazole (25 µg), gentamicin (10 µg), meropenem (10 µg), ofloxacin (5 µg), tetracycline (30 µg) and ticarcillin/clavulanic acid (75/10 µg) according to the method described by Clinical Laboratory Standards Institute (CLSI)^59^. *L. acidophilus* TISTR 2365 was used as a control.

#### Acid-base tolerance

The *F. penangensis* ML061-4 was cultured in TSB overnight. The bacterial cell suspension density was adjusted in 1X PBS (approximately 1 × 10^8^ CFU/ml) prior to evaluation of acid and base tolerant. The acid tolerance was performed in media pH 1.0, 2.0 and 3.0 while the alkaline resistance was done in media containing 0.1, 0.2 and 0.3% (w/v) of bile salt (Himedia^®^)^60^. Survival rate was evaluated by a viable plate count method on TSA following formula:

$${\rm{Survival}}\,{\rm{rate}}\,( \% )=(\log \,\mathrm{CFU}/\mathrm{ml}\,{\rm{of}}\,{\rm{cells}}\,{\rm{survived}}\div\,\log \,\mathrm{CFU}/\mathrm{ml}\,{\rm{of}}\,{\rm{initial}}\,{\rm{cells}}\,{\rm{inoculated}})\times 100.$$

#### Cellular autoaggregation and coaggregation

Autoaggregation was investigated by modification of the protocol described by Valeriano *et al*.^61^. Briefly, the *F. penangensis* ML061-4 was cultured overnight and harvested by centrifugation at 6,000 rpm 4 °C for 5 min. The cell pellets were resuspended in 1X PBS and adjusted equivalent to 0.1 at OD_600_ (OD_i_) and undisturbed at room temperature for 48 hr. The upper suspension fluid was measured the OD_600_ (OD_t_) at 1, 2, 4, 6, 12, 24, 36 and 48 hr. *L. acidophilus* TISTR 2365, a well-known probiotic strain, was used as a control. The autoaggregation percentage was calculated according to the equation:

$${\rm{Autoaggregation}}\,( \% )=[1-({{\rm{OD}}}_{{\rm{t}}}/{{\rm{OD}}}_{{\rm{i}}})]\times 100.$$

The coaggregation against *E. coli* ATCC 25922 and *E. coli* O157:H7 DMST 12743 was carried out^62^. Cell suspensions of *F. penangensis* ML061-4 and test pathogenic bacteria were prepared to 1 × 10^9^ CFU/ml each. After that, the strain ML064-1 was mixed with each test pathogenic bacteria (1 × 10^8^ CFU/ml final concentration of each). The upper suspension fluid was measured the absorbance at 600 nm. The percentage of coaggregation at 0, 1, 2, 4, 6, 12, 24, 36 and 48 hr was calculated according to the equation:

$${\rm{C}}{\rm{o}}{\rm{a}}{\rm{g}}{\rm{g}}{\rm{r}}{\rm{e}}{\rm{g}}{\rm{a}}{\rm{t}}{\rm{i}}{\rm{o}}{\rm{n}}\,({\rm{ \% }})=\{[({{\rm{A}}}_{{\rm{x}}}+{{\rm{A}}}_{{\rm{y}}}/2)-{\rm{A}}({\rm{x}}+{\rm{y}})]/({{\rm{A}}}_{{\rm{x}}}+{{\rm{A}}}_{{\rm{y}}}/2)\}\times 100.$$

A_x_ and A_y_ represented absorbance of each isolate and A(x + y) demonstrated absorbance of mixture.

#### Cell surface hydrophobicity

Cellular hydrophobicity was determined by measuring the bacterial cell adhesion to hydrocarbon according to the protocol described by Meidong *et al*.^27^. In brief, the bacterial cell culture of *F. penangensis* ML061-4 was incubated overnight, harvested by centrifugation and washed twice by 1X PBS. The cell pellet was resuspended in 1X PBS and adjusted equivalent to 0.1 at OD_600_ (A_intial_). The *F. penangensis* ML061-4 suspension was mixed with xylene. The mixture was undisturbed for separation of organic and aqueous phases at room temperature for 30 min. Afterwards, the final absorbance of aqueous phase (A_final_) was measured. *L. acidophilus* TISTR 2365 was used as a control. The percentage of surface hydrophobicity was calculated according to the equation:

$${\rm{Surface}}\,{\rm{hydrophobicity}}\,( \% )=[({{\rm{A}}}_{{\rm{intial}}}-{{\rm{A}}}_{{\rm{final}}})/{{\rm{A}}}_{{\rm{intial}}}]\times 100.$$

#### Bacterial adhesion, competition and exclusion

The bacterial adherence assay was performed following the method described by Llanco *et al*.^63^ with modifications. Briefly, the African green monkey kidney cell line (Vero cell) was grown in Dulbecco’s modified eagle medium (DMEM) (Gibco®, Life Technologies^TM^, UK) supplemented with penicillin/streptomycin and fetal bovine serum (FBS) (Sigma-Aldrich™) and incubated at 37 °C in a CO_2_ incubator. The Vero cells were cultivated in 6-well plate and adjusted equivalent approximately 1 × 10^5^ cell/ml in DMEM with FBS, incubated at 37 °C in a CO_2_ incubator for 18-24 hr and washed three times with 1X PBS afterwards. Subsequently, the suspended ML061-4 cells (1 × 10^8^ CFU/ml) and DMEM were added into each well prior to incubation for 1 hr and washed three times with 1X PBS. The Vero cells were methanol fixed for 3–5 min and stained with Giemsa stain for 10–15 min. *L. acidophilus* TISTR 2365 and *L. plantarum* FM03-1 (GenBank accession no. MF599378), known adhesive strains, were used as controls. Morphological alterations were observed under light microscope and percentage of adhesion to cell line was calculated.

Bacterial competition and competitive exclusion of *E. coli* ATCC 25922 and *E. coli* O157:H7 DMST 12743 by strain ML061-4 was investigated by the method described by Boudeau *et al*.^64^ with modification. In competition experiments, the strain ML064-1 and *E. coli* strain were added together to the cell monolayer (1 × 10^8^ CFU/ml final concentration of each) and incubated for 1 hr. For competitive exclusion, the Vero cell monolayer was first incubated with *E. coli* for 1 hr, washed the non-adherent bacteria three times with 1X PBS, added the strain ML064-1 and incubated for 3 hr.

### Statistical analysis

Student’s t-test was performed to calculate statistical significance of cellular autoaggregation, coaggregation and cell surface hydrophobicity. The bacterial adhesion, competition and competitive exclusion were statistically evaluated using analysis of variance (ANOVA) with SPSS 22.0 and Duncan’s multiple range tests. Significance levels were defined using p < 0.05.

## Conclusions

Leaves of Assam tea or Miang (*Camellia sinensis* var. *assamica*) contained diverse microorganisms including lactic acid bacteria that might participate in Miang fermentation. *F. penangensis* ML061-4 isolated from Assam tea leaves, first report in Thailand, exhibited some biological properties indicating the high potential for using as a probiotic. The *rpoB* gene was first used as a tool for effective identification of bacteria in the genus *Floricoccus*.

## Supplementary information

Supplementary Information

## References

[1] Chuah LO (2017). *Floricoccus tropicus* gen. nov., sp. nov. and *Floricoccus penangensis* sp. nov. isolated from fresh flowers of durian tree and hibiscus. Int J Syst Evol Microbiol.

[2] Beye M, Fahsi N, Raoult D, Fournier PE (2018). Careful use of 16S rRNA gene sequence similarity values for the identification of *Mycobacterium* species. New Microbes New Infect.

[3] Jo JH, Kennedy EA, Kong HH (2016). Research techniques made simple: Bacterial 16S ribosomal RNA gene sequencing in cutaneous research. J Investig Dermatol.

[4] Poretsky R, Rodriguez-R LM, Luo C, Tsementzi D, Konstantinidis KT (2014). Strengths and limitations of 16S rRNA gene amplicon sequencing in revealing temporal microbial community dynamics. Plos One.

[5] Drancourt M, Roux V, Fournier. PE, Raoult D (2004). *rpoB* gene sequence-based identification of aerobic Gram-positive cocci of the genera *Streptococcus*, *Enterococcus*, *Gemella*, *Abiotrophia*, and *Granulicatella*. J Clin Microbiol.

[6] Mollet C, Drancourt M, Raoult D (1997). *rpoB* sequence analysis as a novel basis for bacterial identification. Mol Microbiol.

[7] Okada S, Daengsubha W, Uchimura TAI, Ohara N, Kozaki M (1986). Flora of lactic acid bacteria in miang produced in northern Thailand. J Gen Appl Microbiol.

[8] Khanongnuch C, Unban K, Kanpiengjai A, Saenjum C (2017). Recent research advances and ethno-botanical history of miang, a traditional fermented tea (*Camellia sinensis* var. *assamica*) of northern Thailand. J Ethn Foods.

[9] Kanpiengjai A, Chui-Chai N, Chaikaew S, Khanongnuch C (2016). Distribution of tannin-‘tolerant yeasts isolated from Miang, a traditional fermented tea leaf (*Camellia sinensis* var. *assamica*) in northern Thailand. Int J Food Microbiol.

[10] FAO/WHO. Report of a Joint Food and Agriculture Organization of the United Nations and World Health Organization (FAO/WHO) expert consultation on guidelines for the evaluation of probiotics in food, http://www.who.int/foodsafety/fs_management/en/probiotic_guidelines.pdf (2002).

[11] Kerry RG (2018). Benefaction of probiotics for human health: A review. J Food Drug anal.

[12] Papadimitriou K, Pot B, Tsakalidou E (2015). How microbes adapt to a diversity of food niches. *Curr Opin*. Food Sci.

[13] Klayraung S, Viernstein H, Sirithunyalug J, Okonogi S (2008). Probiotic properties of Lactobacilli isolated from Thai traditional food. Sci Pharm.

[14] Perez RH, Zendo T, Sonomoto K (2014). Novel bacteriocins from lactic acid bacteria (LAB): various structures and applications. Microb Cell Fact.

[15] Stiles ME, Holzapfel WH (1997). Lactic acid bacteria of foods and their current taxonomy. Int J Food Microbiol.

[16] Kawakami M, Chairote G, Kobayashi A (1987). Flavor constituents of pickled tea, Miang, in Thailand. Agric Biol Chem.

[17] Karak T (2015). Major soil chemical properties of the major tea-growing areas in India. Pedosphere.

[18] Kämpfer P, Martin K, Glaeser SP (2013). *Lysinibacillus contaminans* sp. nov., isolated from surface water. Int J Syst Evol Microbiol.

[19] Vuuren SV, Williams VL, Sooka A, Burger A, der Haar LV (2014). Microbial contamination of traditional medicinal plants sold at the Faraday *muthi* market, Johannesburg, South Africa. S Afr J Bot.

[20] Román-Ponce B (2016). *Kocuria arsenatis* sp. nov., an arsenic-resistant endophytic actinobacterium associated with *Prosopis laegivata* grown on high-arsenic-polluted mine tailing. Int J Syst Evol Microbiol.

[21] Prakash O (2014). Description of *Micrococcus aloeverae* sp. nov., an endophytic actinobacterium isolated from *Aloe vera*. Int J Syst Evol Microbiol.

[22] Sunar K, Dey P, Chakraborty U, Chakraborty B (2015). Biocontrol efficacy and plant growth promoting activity of *Bacillus altitudinis* isolated from Darjeeling hills, India. J Basic Microbiol.

[23] Evans R (2017). Defining the functional traits that drive bacterial decomposer community productivity. ISME J.

[24] Qiao J (2017). Addition of plant-growth-promoting *Bacillus subtilis* PTS-394 on tomato rhizosphere has no durable impact on composition of root microbiome. BMC Microbiol.

[25] Jayanthi N, Ratna SM (2015). *Bacillus clausii* - The probiotic of choice in the treatment of diarrhoea. J Yoga Phys Ther.

[26] Elshaghabee FMF, Rokana N, Gulhane RD, Sharma C, Panwar H (2017). *Bacillus* as potential probiotics: status, concerns, and future perspectives. Front Microbiol.

[27] Meidong R (2017). A novel probiotic *Bacillus siamensis* B44v isolated from Thai pickled vegetables (*Phak-dong*) for potential use as a feed supplement in aquaculture. J Gen Appl Microbiol.

[28] Paz A, Outeiriño D, de Souza Oliveira RP, Domínguez JM (2018). Fed-batch production of vanillin by *Bacillus aryabhattai* BA03. New Biotechnol.

[29] Chaves F, García-Álvarez M, Sanz F, Alba C, Otero JR (2005). Nosocomial spread of a *Staphylococcus hominis* subsp. *novobiosepticus* strain causing sepsis in a neonatal intensive care unit. J Clin Microbiol.

[30] Szewczyk EM, Nowak T, Cieślikowski T, Różalska M (2009). Potential role of *Staphylococcus cohnii* in a hospital environment. Microb Ecol Health Dis.

[31] Barros EM, Ceotto H, Bastos MCF, dos Santos KRN, de Marval MG (2011). *Staphylococcus haemolyticu*s as an important hospital pathogen and carrier of methicillin resistance genes. J Clin Microbiol.

[32] Chaikaew S (2017). Diversity of lactic acid bacteria from Miang, a traditional fermented tea leaf in northern Thailand and their tannin-tolerant ability in tea extract. J Microbiol.

[33] Gramza-Michałowska A (2016). Antioxidative potential, nutritional value and sensory profiles of confectionery fortified with green and yellow tea leaves (*Camellia sinensis*). Food Chem.

[34] Li YC (2016). Variations of rhizosphere bacterial communities in tea (*Camellia sinensis* L.) continuous cropping soil by high-throughput pyrosequencing approach. J Appl Microbiol.

[35] Nancib A, Nancib N, Boudrant J (2009). Production of lactic acid from date juice extract with free cells of single and mixed cultures of *Lactobacillus casei* and *Lactococcus lactis*. World J Microbiol Biotechnol.

[36] Kankaanpää PE, Salminen SJ, Isolauri E, Lee YK (2001). The influence of polyunsaturated fatty acids on probiotic growth and adhesion. FEMS Microbiol Lett.

[37] Khamis A, Colson P, Raoult D, Scola BL (2003). Usefulness of *rpoB* gene sequencing for identification of *Afipia* and *Bosea* species, including a strategy for choosing discriminative partial sequences. Appl Environ Microbiol.

[38] Konings WN (1997). The role of transport processes in survival of lactic acid bacteria. Energy transduction and multidrug resistance. Antonie Van Leeuwenhoek.

[39] Papadimitriou K (2016). Stress physiology of lactic acid bacteria. Microbiol Mol Biol Rev.

[40] Hassan M, Kjos M, Nes IF, Diep DB, Lotfipour F (2012). Natural antimicrobial peptides from bacteria: characteristics and potential applications to fight against antibiotic resistance. J Appl Microbiol.

[41] Trip H, Mulder NL, Lolkema JS (2012). Improved acid stress survival of *Lactococcus lactis* expressing the histidine decarboxylation pathway of *Streptococcus thermophilus* CHCC1524. J Biol Chem.

[42] Menconi A (2014). Identification and characterization of lactic acid bacteria in a commercial probiotic culture. Biosci Microb Food Health.

[43] Teixeira JS (2014). Glutamine, glutamate, and arginine-based acid resistance in *Lactobacillus reuteri*. Food Microbiol.

[44] Marteau P, Minekus M, Havenaar R, Huis JHJ (1997). Survival of lactic acid bacteria in a dynamic model of the stomach and small intestine: Validation and the effects of bile. J Dairy Sci.

[45] Collado MC, Meriluoto J, Salminen S (2008). Adhesion and aggregation properties of probiotic and pathogen strains. Eur Food Res Technol.

[46] Xu H, Jeong HS, Lee HY, Ahn J (2009). Assessment of cell surface properties and adhesion potential of selected probiotic strains. Lett Appl Microbiol.

[47] Silva MS (2017). Probiotic properties of *Weissella cibaria* and *Leuconostoc citreum* isolated from tejuino - A typical Mexican beverage. LWT - Food Sci Technol.

[48] Sherman PM (2005). Probiotics reduce enterohemorrhagic *Escherichia coli* O157:H7- and enteropathogenic *E. coli* O127:H6-induced changes in polarized T84 epithelial cell monolayers by reducing bacterial adhesion and cytoskeletal rearrangements. Infect Immun.

[49] Polak-Berecka M, Waśko A, Paduch R, Skrzypek T, Sroka-Bartnicka A (2014). The effect of cell surface components on adhesion ability of *Lactobacillus rhamnosus*. Antonie Van Leeuwenhoek.

[50] Kanmani P (2013). Synthesis and functional characterization of antibiofilm exopolysaccharide produced by *Enterococcus faecium* MC13 isolated from the gut of fish. Appl Biochem Biotechnol.

[51] Kumari A, Angmo K, Monika & Bhalla TC (2016). Probiotic attributes of indigenous *Lactobacillus* spp. isolated from traditional fermented foods and beverages of north-western Himalayas using *in vitro* screening and principal component analysis. J Food Sci Technol.

[52] Pitcher DG, Saunders NA, Owen RJ (1989). Rapid extraction of bacterial genomic DNA with guanidium thiocyanate. Lett Appl Microbiol.

[53] Weisburg WG, Barns SM, Pelletier DA, Lane DJ (1991). 16S ribosomal DNA amplification for phylogenetic study. J Bacteriol.

[54] Meucci A (2015). *Lactococcus hircilactis* sp. nov. and *Lactococcus laudensis* sp. nov., isolated from milk. Int J Syst Evol Microbiol.

[55] Saitou N, Nei M (1987). The neighbor-joining method: A new method for reconstructing phylogenetic trees. Mol Biol Evol.

[56] Kumar S, Stecher G, Tamura K (2016). MEGA7: Molecular evolutionary genetics analysis version 7.0 for bigger dataset. Mol Biol Evol.

[57] Arroyo E, Enríquez L, Sánchez A, Ovalle M, Olivas A (2014). Scanning electron microscopy of bacteria *Tetrasphaera duodecadis*. Scanning.

[58] Bonev B, Hooper J, Parisot J (2008). Principles of assessing bacterial susceptibility to antibiotics using the agar diffusion method. J Antimicrob Chemo.

[59] Clinical Laboratory Standards Institute. (2012). Performance standards of antimicrobial disc susceptibility tests. CLSI.

[60] Fossi BT (2015). Probiotic properties of lactic acid bacteria isolated from fermented sap of palm tree (*Elaeis guineensis*). J Microbiol Antimicrob.

[61] Valeriano VD, Parungao-Balolong MM, Kang DK (2014). *In vitro* evaluation of the mucin-adhesion ability and probiotic potential of *Lactobacillus mucosae* LM1. J Appl Microbiol.

[62] Buswell CM, Herlihy YM, Marsh PD, Keevil CW, Leach SA (1997). Coaggregation amongst aquatic biofilm bacteria. J Appl Microbiol.

[63] Llanco LA, Nakano V, de Moraes CTP, Piazza RMF, Avila-Campos MJ (2017). Adhesion and invasion of *Clostridium perfringens* type A into epithelial cells. Braz J Microbiol.

[64] Boudeau J, Glasser AL, Julien S, Colombel JF, Darfeuille-Michaud A (2003). Inhibitory effect of probiotic *Escherichia coli* strain Nissle 1917 on adhesion to and invasion of intestinal epithelial cells by adherent-invasive *E. coli* strains isolated from patients with Crohn’s disease. Aliment Pharmacol Ther.

