# Supplementary material for: Partial rpoB Gene Sequencing Identification and Probiotic Potential of Floricoccus penangensis ML061-4 Isolated from Assam Tea (Camellia sinensis var. assamica)
Source: Sci Rep. 2019 Nov 12;9:16561. doi: 10.1038/s41598-019-52979-9 (PMC6851367; doi:10.1038/s41598-019-52979-9)

Supplementary Information

Partial *rpoB* Gene Sequencing Identification and Probiotic Potential of *Floricoccus penangensis* ML061-4 Isolated from Assam Tea (*Camellia sinensis* var. *assamica*)

Patthanasak Rungsirivanich, Angkhana Inta, Yingmanee Tragoolpua and Narumol Thongwai

Legends of Supplementary figures

Supplementary figure S1. The area indicating the Assam tea plant sampling site in Nan province of Thailand.

Supplementary figure S2. Phylogenetic relationships of the bacteria isolates (bold) isolated from Assam tea leaves. The branching pattern was generated using the neighbour-joining tree based on the 16S rRNA gene of bacterial isolates obtained from fresh Assam tea leaves with their closest species. Bootstrap values (expressed as percentages of 1,000 replications). Bar, 0.02 substitutions per nucleotide position. *Escherichia coli* ATCC 43893 (GenBank accession no. HM194886) is presented as an outgroup sequence.

Supplementary figure S1

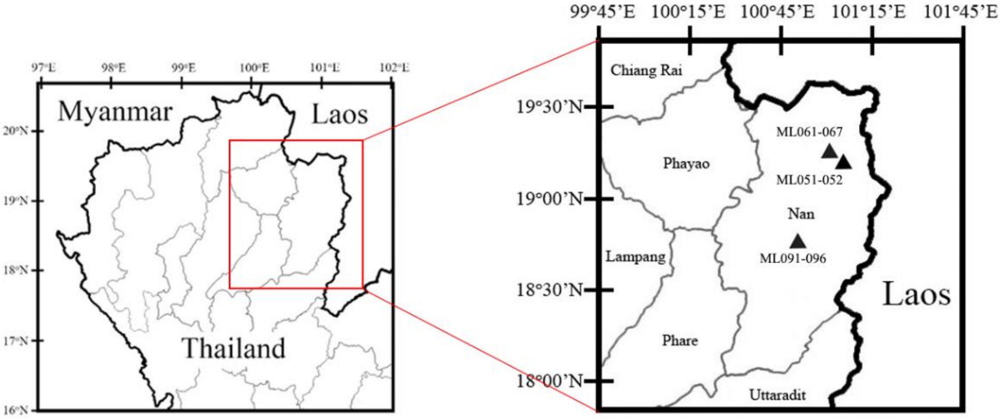

Supplementary figure S2

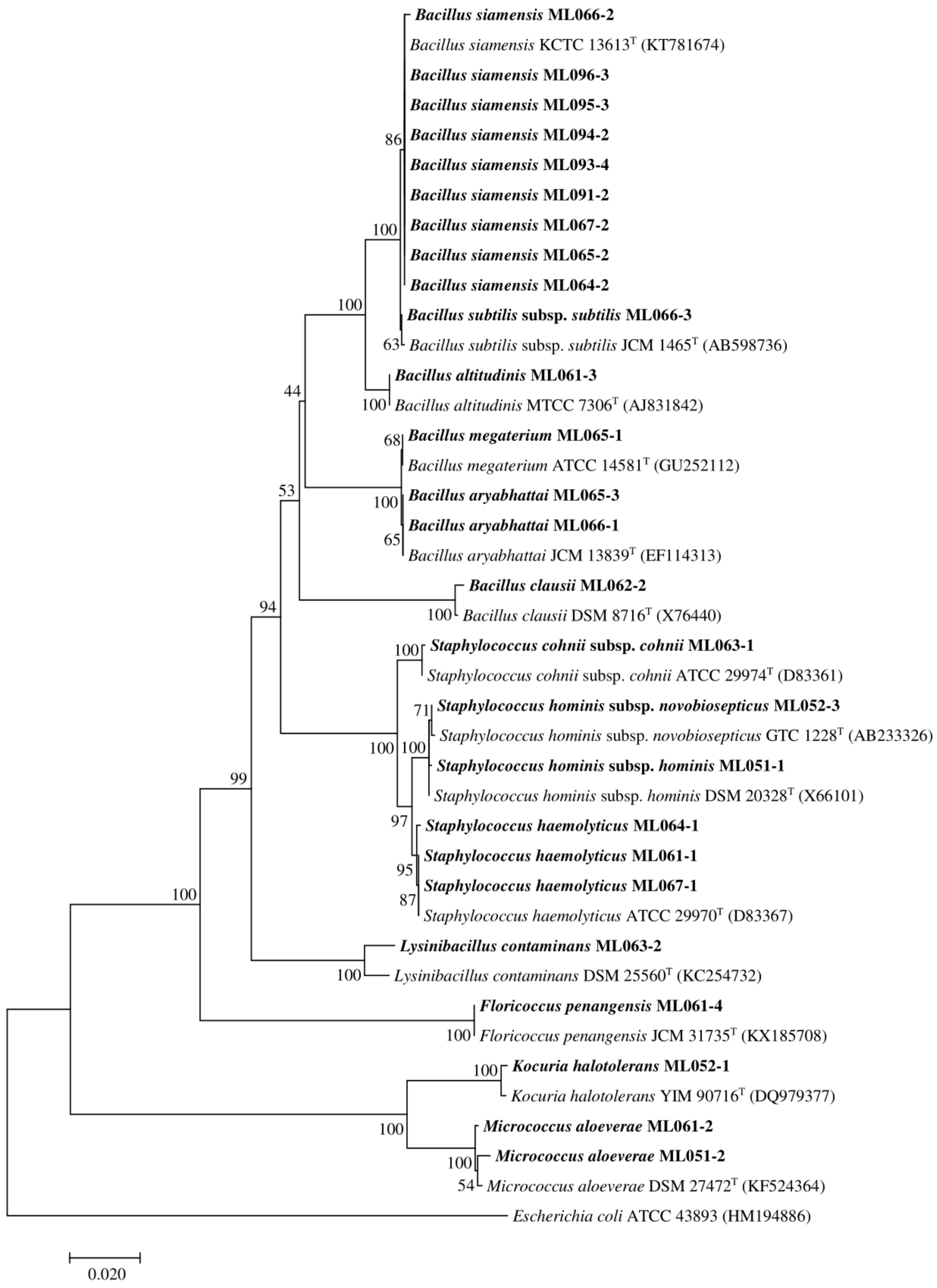

Supplement: Supplementary file 1 — Supplementary Information [file 41598_2019_52979_MOESM1_ESM.pdf]
